# Emotion and cognition in personal and collective work-identity formation: variable- and person-oriented analyses

**DOI:** 10.1016/j.heliyon.2021.e07210

**Published:** 2021-06-04

**Authors:** Ola Nordhall, Igor Knez, Johan Willander

**Affiliations:** Department of Occupational Health Science and Psychology, University of Gävle, SE-801 76 Gävle, Sweden

**Keywords:** Personal work-identity formation, Collective work-identity formation, Emotion, Cognition, Variable- and person-oriented analyses

## Abstract

The aim of this study was to investigate emotional and cognitive processes involved in the formation of personal and collective work-identity by variable- and person-oriented analyses. A digitized questionnaire was answered by 768 participants. In line with an autobiographical (personal) memory view, we showed that: (1) emotional processes positively predicted cognitive processes (variable-oriented analyses), and (2) emotional profile had an effect on cognitive processes (person-oriented analyses), with regard to personal work-identity formation. Regarding collective work-identity formation, and in line with a social-identity and self-categorization perspective, we showed that: (1) cognitive processes positively predicted emotional processes (variable-oriented analyses), and (2) cognitive profile had an effect on emotional processes (person-oriented analyses). Our results indicate that emotion and cognition play different roles in personal- and collective work-identity formation; additionally, suggesting that the theoretical views of both personal and social psychology as well as analyses at different levels should be involved in order to gain a deeper understanding of the phenomenon of people-work bonding.

## 1 Introduction

The concept of work-identity relates to the question of how we categorize and define ourselves in terms of individual and social attributes at work (Cappelli, 2000; Causer and Jones, 1996; Turner et al., 1987). This phenomenon can be divided into *personal* (*I/Me*-descriptions) and *collective* identity (*We*-descriptions). Embracing a strong identity at one level (e.g. personal) does not exclude a similarly strong identity at another level (e.g. collective). This suggests that the two types of work-identity formations are to some extent independent of each other (Brewer and Gardner, 1996; Pate et al., 2009), shaping “functionally independent cognitive structures, leading to separate motivations and influences on work-related satisfaction” (Knez, 2016, p. 1).

Work-identity has been shown to be associated in different ways with a wide range of work- and organization-related behaviors, norms and attitudes, such as work motivation, job satisfaction, in- and extra role behaviors and organizational justice (Lee et al., 2015; Nordhall and Knez, 2018; Nordhall et al., 2020; Riketta, 2005; Riketta and Van Dick, 2005). Associations have also been found with several aspects of self-reported mental health, for example, general mental health, work stress, depression, burnout and exhaustion (see Haslam et al., 2009; Haslam, 2014; Jetten et al., 2012; Nordhall et al., 2018; Steffens et al., 2016).

The psychological formation of work-identity is associated with the phenomenon of self-construal and comprises concepts of self-categorization, identification and autobiographical work-related experiences (Ashforth and Mael, 1989; Brewer and Gardner, 1996; Knez, 2016; Mael and Tetrick, 1992; Turner et al., 1987, see Hogg, 2012 for an overview).

The psychological formation of personal work-identity has broadly been defined as a need to *distinguish* oneself from others (Brewer and Gardner, 1996), “in order to preserve the personal self, the personal story and its memories” (Knez, 2016, pp. 3). However, formation of collective work-identity has been defined in terms of social work-identification involving the knowledge of *belonging* to a certain group “in order to be part of the collective self, the collective story and its memories” (Knez, 2016, p. 3). Such collective work-identity includes a depersonalization of the individual self and an emotional attachment to the group/organization (Tajfel and Turner, 1979; 1986; see Hogg, 2012 for a review).

Work-identification involves *emotional* and *cognitive* processes accounting for the formation of work-identity (Knez, 2016; Millward and Haslam, 2013; Van Knippenberg and Van Schie, 2000).

Emotional and cognitive self-related processes in work-identity formation might be affected by external factors, such as work environment, and organizational and social structures (Day et al., 2006; Gioia et al., 2013; Hogg, 2012). Additionally, emotional and cognitive processes are supposed to be causally related in work-identity formation, i.e. related along a temporal dimension (see Knez, 2014; 2016). Furthermore, we suggest that the employment/organizational time may be one of the main constituents of work-identity formation in that a period of employment may affect our identifications with the occupational work and/or the organization.

In view of this, the aim of the present study was to investigate how different psychological processes, comprising emotion and cognition components, relate to each other in work-identity formation by: (a) *Variable-oriented analysis*, including identity levels/processes involved in work-identity formation; and (b) *Person-oriented analysis*, investigating how individuals group into different clusters/profiles of work-identity formation. As far as we know, these relationships and types of analyses have not been addressed by previous research. However, knowledge concerning emotional and cognitive processes and profiles involved in work-identity formation might be of significant importance for organizational research and organizations per se (see Brown, 2019).

Given that we have not measured the formation of work-identity across time, we will report data on the *momentary set up* of work-identity in subjects with a mean employment time of 14 years (see Method/Participants section). By momentary set up we mean the participants’ present work-identity which they have acquired over the years as employees. The difference between the development of work-identity across time (work-identity formation per se, see Haslam and Ellemers, 2011; Knez 2014; 2016; Riketta, 2005; Van Dick et al., 2004) and the one reported in the present study is that our data report the *momentary set up* of work-identity relatively the period of employment. By this, our data may tentatively suggest how work-identity formation is developed. That is, how the identity-profession relationship may converge into the phenomenon of work-identity across the employment.

### 1.1 Emotion and cognition in work-related personal and collective self/identity

**Figure 1:**
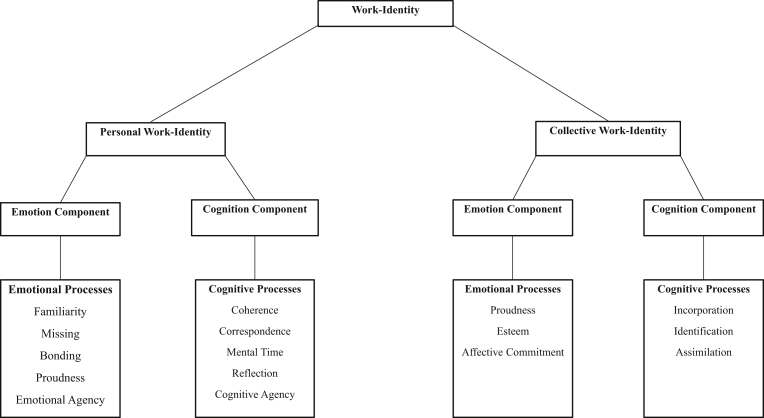
Tentative schematic model of work-identity comprising the two levels of personal and collective work-identity, which involve emotion and cognition components, which in turn contain emotional and cognitive processes, respectively.

Basic psychological processes of emotion and cognition components involved in personal and collective identity formation (see Figure 1) are assumed to interplay with the social contexts of, for example, geographical location (Knez, 2014; Knez et al., 2018a; b) and employment (see Ferris et al., 2018; Johnson et al., 2018; Knez, 2016).

The emotional work-identity stems from our childhood when we develop emotional bonds and attachments, to different people and environments (Knez, 2014). As adults, we expand these bonds including our occupational work as well (Knez, 2016).

The cognitive work-identity concerns the preconditions for all human experience to be organized and structured i.e. cognitively categorized in terms of time/time continuity (past, present, future), and the ability to perceive and reflect upon such memories as belonging to *oneself* (see Knez, 2014; 2016 for these types of arguments).

In line with Knez (2014), Knez (2016) suggested a conceptual model for work-related self/identity, involving emotion and cognition components that account for the people-work bonding phenomena (see also Van Dick and Wagner, 2002). According to this model (Knez, 2014; 2016; see also Knez et al., 2019), personal work-identity may account for personal, autobiographical, work-related experiences (Knez, 2016).

The emotion component of personal work-identity involves processes of work-related familiarity, missing, emotional bonding, proudness and emotional agency (work as part of oneself). These processes indicate an affective closeness/attachment/belonging between an employee and his/her employment (see also Knez, 2014 for this type of argument). The cognition component of personal work-identity includes processes of work-related coherence (continuity in the self-work relation across time), correspondence (adaptive interactions between the self and its working contexts), mental time (temporality in the person-work bonding), reflection (upon ones work-related memories) and cognitive agency (work-related memories as part of oneself) (see Knez, 2016). Knez (2016) additionally assumed that emotion, compared with the cognition component, would temporally precede in work-identity formation (see also Knez, 2014; Knez and Eliasson, 2017; Knez et al., 2018a; b for a similar view). This model was based on structural equation modeling (see Knez, 2014) showing that the model where the emotion component predicted the cognition component had a better data fit, compared to the model where the cognition component predicted the emotion component. In regression terms, the model with best data fit implied that the emotion component might be conceived as a predictor variable and the cognition component as a criterion variable.

Collective, in contrast to personal, work-identity, is supposed to be more of a cognitive entity (Ashforth and Mael, 1989; Harquail and King, 2003) and has been described as “a product of the dialectic relationship between collective, shared cognition on the one hand and socially structured individual cognitions on the other” (Corley et al., 2006, p. 88).

According to the social identity perspective (e.g. Ashforth and Mael, 1989; Tajfel and Turner, 1986) and social categorization theory (Turner et al., 1987) the cognitive bonding to the collective implies perception of oneness and belongingness to e.g. a work organization in terms of organizational membership as incorporated in the self-concept. Such cognitive/perceptual awareness of the organization as part of the self is the essence of organizational identification (Ashforth, 2016; Ashforth and Mael, 1989).

However, collective work-identity can be viewed as multidimensional in its nature comprising both cognitive and emotional components of organizational identification, which has been suggested by theoretical and empirical accounts (see Ellemers et al., 2004; Johnson et al., 2018; Kreiner and Ashforth, 2004; Van Dick et al., 2004; Xenikou, 2014). The cognition component is defined in terms of a person's knowledge or awareness of a particular social categorization and interdependence while the emotion component is defined in terms of a person's affective involvement in the organization (Ellemers et al., 1999, 2004; Xenikou, 2014).

In line with Nordhall and Knez (2018) and Nordhall et al. (2018), the cognition component of collective work-identity comprises processes of incorporation (of peoples’ organizational perceptions), identification (“we” descriptions of the organization) and assimilation (of organizational successes). The emotion component of collective work-identity involves processes of proudness (of organizational belonging), esteem (of organizational belonging), and affective commitment (to the organization) (see also Ashforth and Mael, 1989; Harquail and King, 2003; Mael and Ashforth, 1992; Mael and Tetrick, 1992; Van Knippenberg and Sleebos, 2006). In contrast to personal work-identity formation, the cognition component of collective work-identity formation is supposed to temporally precede and affect the emotional one (see Mael and Ashforth, 1992; Van Knippenberg and Sleebos, 2006). For example Van Dick et al. (2004) in two cross-validated samples indicated that collective identification involves a cognitive process leading the employee to consider him-/herself as a member of an organization, which, in turn, activates other dimensions such as affective commitment/reactions of the identification mechanism (Van Dick et al., 2004). In line with Knez (2014; 2016) and in regression terms, this suggests that the cognition component might be defined as a predictor and the emotion component as a criterion variable.

In the present study, the concept of work-identity formation emanates from two theoretical views (Millward and Haslam, 2013; Reichers, 1985; Van Knippenberg and Van Schie, 2000): (1) an autobiographical memory perspective (e.g., Knez, 2014; 2016; Knez, 2017; Knez et al., 2017; Knez and Nordhall, 2017); and (2) a social identity perspective (e.g. Ashforth and Mael, 1989). In other words, we broaden the multiple focus of the work-identity formation concept by suggesting that the phenomenon of people-work bonding can be treated from both an autobiographical memory perspective (*individual –* personal work-related self/identity) and an organizational/workgroup perspective (*social* – collective work-related self/identity).

### 1.2 Work-identity formation: variable- and person-oriented analyses

As outlined above, previous research has investigated effects of work-identity formation in relation to personal/individual, relational/interpersonal and collective/organizational types of work-identity classifications (Hogg and Terry, 2000; Knez, 2016). However, there has been less focus on the different psychological processes (e.g. missing, proudness, coherence, correspondence, incorporation, assimilation, esteem and affective commitment) that make up the components (e.g. emotion and cognition) of work-identity (e.g., Nordhall and Knez, 2018; Nordhall et al., 2018; Nordhall et al., 2020).

Thus, the focus of the present study was to investigate how different psychological processes, comprising emotion and cognition components, relate to each other in work-identity formation. This means: (1) Variable-oriented analysis, including identity levels (personal vs. collective), components (emotion vs. cognition) or processes involved in work-identity formation (analyzed, for example, by a linear regression analysis) (see Bergman, 2000; Bergman et al., 2000; Magnusson, 2000); (2) Person-oriented analysis, investigating how individuals group into different clusters (i.e., profiles) along certain central processes involved in work-identity formation (for examples, see above) and how differences between such work-identity profiles associate with other central psychological processes involved in work-identity formation (for examples, see above). Such a person-oriented analysis may enhance the understanding of work-identity formation (Day et al., 2006; Dutton et al., 2010; Gioia et al., 2013) as it entails a holistic analysis of the individual as an organizing whole, where individuals may be grouped into different clusters with different work-identity profiles (i.e. combinations) of a set of seemingly compartmentalized factors, e.g. processes. This is in contrast to a variable-oriented analysis where each variable is treated as a separate entity, thereby missing the holistic integration/combination, i.e. interaction, of different psychological processes involved in work-identity formation (see Bergman, 2000; Bergman et al., 2000; Magnusson, 2000).

A person-oriented analysis, using cluster analysis, has been carried out in many studies (e.g. see Clatworthy et al., 2005; Kim et al., 2018; Lewandowski et al., 2018; Lövdén et al., 2005; Stenlund et al., 2018) but has been less common in research on work-identity formation. In these relatively few studies, individuals have been clustered along *indicators* of their professional identity e.g. satisfaction, commitment, interests, competences, values and goals, social embeddedness, and behavioral involvement in one's work (see Canrinus et al., 2011; Endedijk et al., 2017; Groth et al., 2017; Pillen et al., 2013). This is in contrast to clustering along self-related processes (see Knez, 2014; 2016) which was the case in the present study.

In view of this, individuals may differ on the dimensions of strength and consistency in their work-identity, where different identity processes may be involved. This means that individuals may vary in how strongly they identify with their work (i.e. strength) and the extent to which they have similar levels of identification across the different psychological processes (i.e. consistency) involved in work-identity formation (see Canrinus et al., 2011; Strachan et al., 2009; Welbourne and Paterson, 2017; Wright, 2009). This implies that individuals may be positioned along *four* combinations of strength and consistency in personal and collective work-identity. Accordingly, individuals are assumed to group into four clusters based on their combination of strength and consistency in work-identity processes, see Table 1 for details.

**Table 1:** Cell matrix for the four different strength [strong (S) vs. weak (W)] and consistency [consistent (C) vs. inconsistent (I)] combinations in personal and collective work-identity formation.

Consistency	Strength	
	Strong (S)	Weak (W)
Consistent (C)	SC	WC
Inconsistent (I)	SI	WI

## 2 Aims

**Figure 2:**
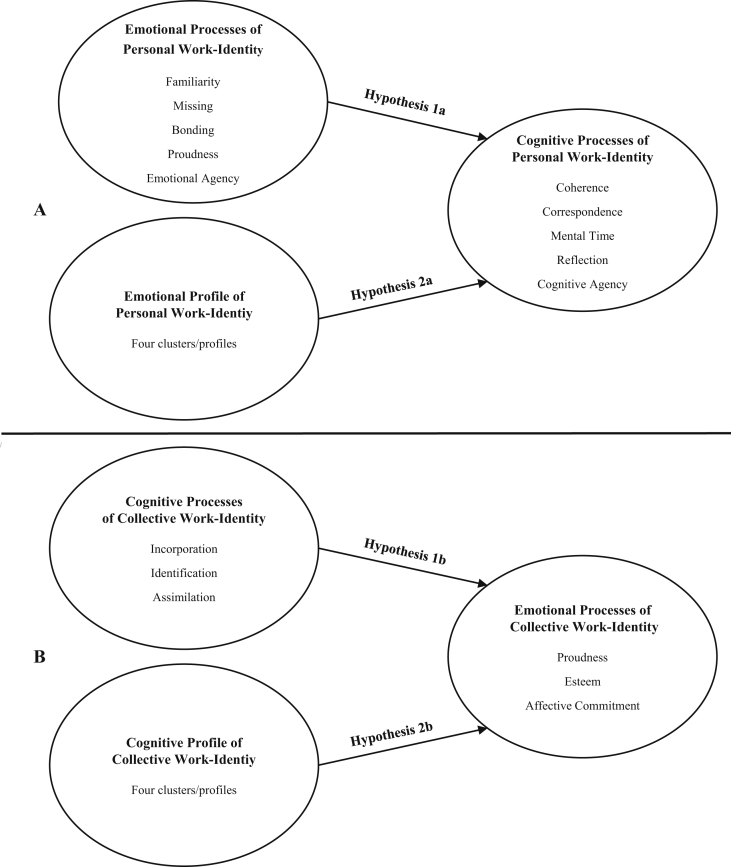
Aims and hypotheses details of *personal* work-identity (A) and *collective* work-identity (B) formation, including variable-oriented analyses (Hypothesis 1a and 1b) and person-oriented analyses (Hypothesis 2a and 2b).

Given the above, the aims of the present study were to investigate: 1a) emotional processes predicting cognitive processes involved in formation of *personal* work-identity; 1b) cognitive processes predicting emotional processes involved in formation of *collective* work-identity; 2a) effects of emotional profile on cognitive processes in *personal* work-identity formation; and 2b) effects of cognitive profile on emotional processes in *collective* work-identity formation. For details, see Figure 2.

### 2.1 Hypotheses

***Hypothesis 1*** (variable-oriented analyses): We hypothesized that: 1a) Emotional processes (predictor variables) will positively predict cognitive processes (criterion variables) of *personal* work-identity; and 1b) Cognitive processes (predictor variables) will positively predict emotional processes (criterion variables) of *collective* work-identity (see Figure 2 and Design and analyses section below for more details).

***Hypothesis 2*** (person-oriented analyses). We hypothesized: 2a) Effects of emotional profile (independent variable) on cognitive processes (dependent variables) of *personal* work-identity; 2b) Effects of cognitive profile (independent variable) on emotional processes (dependent variables) of *collective* work-identity (see Figure 2 and Design and analyses section below for more details).

## 3 Method

The present study is a part of a larger research project on work-identity. Accordingly, the method section is consonant, in general terms, with previous publications within this project (Nordhall and Knez, 2018; Nordhall et al., 2018, 2020).

### 3.1 Participants

The participants consisted of members (teachers) of the Swedish trade union, “The National Union of Teachers” (in Swedish “Lärarnas Riksförbund”). The participants represented eleven different local branches of the union and worked in the southern and the middle part of Sweden. The response comprised 768 (26%) questionnaires that were returned (out of 2,905 individuals that received the questionnaire). Due to lack of information, no analysis of the non-respondents could be performed. For the demographical data of the participants in the present study and a comparison sample of Swedish teachers (Skolverket, n.d.), see Table 2, which indicates that the participants in the present study are representative of the Swedish teacher population.

**Table 2:** Demographical data for the participants in the present study (N = 768) and for a comparison sample of Swedish teachers (N = 133 741) based on statistics from year 2016.

Demographical data	Participants (N = 768)	Comparison sample (N = 133 741)
Teachers/Educational function (%)	99.0	100
Permanent employment (%)	99.0	85.0
Non-private sector (%)	92.1	89.4
Full-time employment (%)	80.5	73.0
Gender/females (%)	75.5	76.0
Education/University studies (%)	68.0	81.3
Age (Mean years and SD)	46.3 (SD = 10.1)	46.0 (SD = 11.4)
Mean employment time within the organization (years)	14	13.5

### 3.2 Measures

#### 3.2.1 Work-identity

To measure personal work-identity we used “Work-related Self Measure”, developed by Knez (2016; see also Knez and Eliasson, 2017; Knez et al. 2019). The instrument comprises ten statements measuring emotional and cognitive processes involved in personal work-identity formation: Emotional: (1) Familiarity: “I am keenly familiar with my work”; (2) Missing: “I miss it when I'm not there.”; (3) Bonding: “I have strong ties to my work."; (4) Proudness: “I am proud of my work.”; (5) Emotional Agency: “My work is a part of me.”. Cognitive: (6) Coherence: “I have had a personal relation with my work over a long period.”; (7) Correspondence: “There is a link between my work and my current life."; (8) Mental Time: “Mentally I can travel back and forth in time to my work when I think about it.”; (9) Reflection: “I can reflect on the memories of my work”; (10) Cognitive Agency: “My thoughts and memories about my work are part of me.”. The participants responded to the statements on a five-point Likert scale, ranging from 1 (completely disagree) to 5 (completely agree). The Cronbach alpha (α) value was .86 for personal work-identity, .75 for emotional and .84 for cognitive components respectively in the present study, showing acceptable-good internal consistency (see DeVillis, 2003). Previous research (e.g. Knez et al., 2019) has reported Cronbach alpha (α) of .86 for personal work-identity (index). Construct validity statistics for the personal work-identity construct/measure, have been reported by Nordhall and Knez (2018), showing an acceptable data fit of Chi^2^ = 188.57, df = 28 (p = .000), CFI = .95 and RMSEA = .08 (see Byrne, 2016).

Collective work-identity was measured by the “Identification with a Psychological Group Scale” (Mael and Ashforth, 1992; Mael and Tetrick, 1992; Riketta, 2005), theoretically grounded in Social Identity Theory (Tajfel & Turner, 1979, 1986), and Self-Categorization Theory (Hogg and Terry, 2000). Six statements with a five-point Likert scale, ranging from 1 (completely disagree) to 5 (completely agree) are included in this measure. In line with the conceptual model of Knez (2014; 2016), which distinguishes between emotion and cognition components of work-identity (Jackson et al., 2006; Knez, 2016), three items of the “Identification with a Psychological Group Scale” (Mael and Ashforth, 1992) were categorized as measuring the cognitive processes; (1) Incorporation: “I am very interested in what others think about the organization.”; (2) Identification: “When I talk about this organization, I usually say wé rather than ‘they’.”; (3) Assimilation: “This organization’s successes are my successes.”) and the emotional processes: (4) Proudness: “When someone criticizes my organization, it feels like a personal insult.”; (5) Esteem: “When someone praises the organization it feels like a personal compliment.”; (6) Affective commitment: “If a story in the media criticized the organization, I would feel embarrassed.”), respectively. This was carried out in line with Mael and Ashforth's (1992) suggestions (also supported by Hogg, 2012; Tajfel, 1972; 1978). The different scales showed the following Cronbach alphas (α): .87 for collective work-identity, .78 for emotional- and .77 for cognitive component, indicating good internal consistency (see DeVillis, 2003). Previous research (e.g. Mael and Ashforth, 1992) has reported Cronbach alphas (α) of .81–.87 for collective work-identity (index). In addition, and as above, Nordhall and Knez (2018) reported an acceptable construct validity data fit of Chi2 = 64.09, df = 7 (p = .000), CFI = .97 and RMSEA = .10 for the collective work-identity concept/measure (see Byrne, 2016).

### 3.3 Procedure

The chairpersons of eleven municipal associations, from “The Swedish National Union of Teachers”, were asked to invite their members to participate in a survey about work-identity. This procedure was necessary as Swedish law restricted the chairpersons from giving out their members’ email addresses. A web-link to the questionnaire was thus distributed to the members by the chairpersons. The purpose of the project was described in a covering letter accompanying the questionnaires that also informed the participants that anonymity and confidentiality were assured, and that completion of the questionnaire indicated their consent to participate voluntarily. The participants were asked to fill in their name and address after they had completed the questionnaire if they wanted to receive a cinema ticket as compensation for their participation. They received information that no-one other than the researchers of the present study would have access to their names and addresses

In this study, we analyzed data related to emotional and cognitive processes involved in personal and collective work-identity formation.

Finally, an ethical application was reviewed and approved by the Swedish Regional Ethical Review Board of Uppsala (Dnr 2015/423).

### 3.4 Design and analyses

The aims and hypotheses 1a-b (variable-oriented analysis), were investigated by regression model statistics (see Bergman, 2000; Bergman et al., 2000; Magnusson, 2000). Two types of multiple regression analyses, using IBM SPSS Statistics 24, including the following predictors and criterion variables, were performed in order to investigate Hypothesis 1a: the role of the emotional process (predictors) in predicting the cognitive processes (criterion variables) in personal work-identity formation, and Hypothesis 1b: the role of the cognitive processes (predictors) in predicting the emotional processes (criterion variables) in collective work-identity formation.

Concerning the aims and hypotheses 2a-b (person-oriented analysis), individuals were first clustered (see Bergman, 2000; Bergman et al., 2000; Magnusson, 2000; Romesburg, 1984), using IBM SPSS Statistics 24, hierarchical clustering with Ward's method and squared Euclidean distance, in order to obtain different clusters, i.e. profiles, in personal and collective work-identity. The emotional process of personal work-identity (see Aim 2a and Measures) and the cognitive processes of collective work-identity (see Aim 2b and Measures), respectively were used to form clusters.

For design details, see also Figure 2 above.

In line with Aims and Hypotheses 2a-b, two types of MANOVA, using IBM SPSS Statistics 24, including the following independent- and dependent variables, were performed in order to investigate Hypothesis 2a) the effects of emotional profile (independent variable with four levels i.e. profiles) on the cognitive processes (dependent variables) of personal work-identity formation, and Hypothesis 2b) the effects of cognitive profile (independent variable with four levels i.e. profiles) on the emotional processes (dependent variables) of collective work-identity formation.

Level of significance (alpha) was .05 in all analyses, except for the univariate analyses in the MANOVAs where Bonferroni adjustment of alpha level means *p* < .01 due to dividing the original alpha level of .05 by the number of analyses that were intended (see Tabachnick and Fidell, 2012).

Note: In the present study “prediction” and “predict” (Hypothesis 1a&b) do not imply a causal prediction but, in a statistically/mathematical sense, implying prediction of a criterion value given a specific predictor value on a regression line. Also, the “effects” (Hypothesis 1&b) relates to the use of a quasi-experimental design with independent and dependent variables, however not indicating causation in equally strong sense as in studies using experimental design. For these types of arguments see Knez et al. (2019, 2018a; b) and Tabachnick and Fidell (2012).

## 4 Results

First, we report the bivariate correlations, N, mean and standard deviation statistics for all variables included in the regression analyses (see Table 3). Then, we report the results for our hypotheses and the types of regression analyses related to each of the two hypotheses respectively (see section Design and Analyses), i.e. the variable-oriented analysis.

**Table 3:** Bivariate correlations (r), *N*, mean (*M*) and standard deviation (*SD*) statistics for all variables included in the regression analyses. Emotional processes of personal work-identity: Familiarity; Missing; Bonding; Proudness; Emotional Agency. Cognitive process of personal work-identity: Coherence; Correspondence; Mental Time; Reflection; Cognitive Agency. Cognitive processes of collective work-identity: Incorporation; Identification; Assimilation, and Emotional processes of collective work-identity: Proudness; Esteem; Affective Commitment.

	N	M	SD	1	2	3	4	5	6	7	8	9	10	11	12	13	14	15
1. Familiarity	767	4.44	.69															
2. Missing	767	2.31	1.15	.100∗∗														
3. Bonding	767	3.60	1.08	.256∗∗	.462∗∗													
4. Proudness	767	4.12	.93	.249∗∗	.341∗∗	.478∗∗												
5. Emotional Agency	767	3.65	1.08	.134∗∗	.434∗∗	.649∗∗	.490∗∗											
6. Coherence	767	3.55	1.14	.326∗∗	.340∗∗	.628∗∗	.392∗∗	.688∗∗										
7. Correspondence	767	3.27	1.18	.143∗∗	.307∗∗	.432∗∗	.203∗∗	.507∗∗	.556∗∗									
8. Mental Time	767	3.65	1.14	.094∗∗	.228∗∗	.311∗∗	.155∗∗	.397∗∗	.419∗∗	.479∗∗								
9. Reflection	767	3.91	.94	.157∗∗	.250∗∗	.361∗∗	.244∗∗	.393∗∗	.446∗∗	.441∗∗	.556∗∗							
10. Cognitive Agency	767	3.70	1.07	.132∗∗	.287∗∗	.466∗∗	.204∗∗	.554∗∗	.570∗∗	.568∗∗	.545∗∗	.681∗∗						
11. Incorporation	767	2.81	1.11	.075∗	.194∗∗	.181∗∗	.184∗∗	.152∗∗	.163∗∗	.127∗∗	.074∗	.121∗∗	.170∗∗					
12. Identification	767	3.12	1.26	.055	.131∗∗	.201∗∗	.146∗∗	.207∗∗	.185∗∗	.120∗∗	.096∗∗	.117∗∗	.198∗∗	.444∗∗				
13. Assimilation	767	2.97	1.15	.041	.182∗∗	.272∗∗	.220∗∗	.253∗∗	.198∗∗	.134∗∗	.129∗∗	.116∗∗	.199∗∗	.475∗∗	.640∗∗			
14. Proudness	767	2.29	1.14	-.018	.156∗∗	.180∗∗	.082∗	.191∗∗	.187∗∗	.113∗∗	.116∗∗	.103∗∗	.198∗∗	.452∗∗	.460∗∗	.503∗∗		
15. Esteem	767	2.72	1.15	.061	.181∗∗	.235∗∗	.213∗∗	.215∗∗	.209∗∗	.121∗∗	.108∗∗	.121∗∗	.180∗∗	.467∗∗	.621∗∗	.801∗∗	.593∗∗	
16. Affective Commitment	767	2.75	1.26	-.020	.159∗∗	.174∗∗	.073∗	.190∗∗	.186∗∗	.118∗∗	.153∗∗	.175∗∗	.202∗∗	.411∗∗	.404∗∗	.491∗∗	.547∗∗	.505∗∗

∗∗ Correlation is significant at the .01 level (2-tailed).

∗ Correlation is significant at the .05 level (2-tailed).

The regression analyses below did not indicate multicollinearity effects, showing tolerance values of >.10, range .410–.910 and all VIF (variance inflation factor) < 10, range 1.100–2.010 (see Menard, 1995; Myers, 1990; Tabachnick and Fidell, 2012).

Third, we report the results for the aims and hypothesis associated with the cluster analysis and the MANOVA, i.e. the person-oriented analysis.

### 4.1 Variable-oriented analysis

#### 4.1.1 Emotional processes predicting cognitive processes of personal work-identity

In line with Hypothesis 1a, the emotional processes significantly predicted each of the cognitive processes in personal work-identity with an explained variance of 57% for Coherence, 29% for Correspondence, 17% for Mental Time, 18% for Reflection and 34% for Cognitive Agency. Emotional Agency was by far strongest associated while Proudness, negatively, or not at all, associated with each of the five cognitive processes of personal work-identity. Familiarity, Missing and Bonding showed mixed relationships in association strength and slope direction regarding the five cognitive processes of personal work-identity (see Table 4 for details).

**Table 4:** The emotional processes (Familiarity, Missing, Bonding, Proudness and Emotional Agency), predicting the cognitive processes (Coherence, Correspondence, Mental Time, Reflection and Cognitive Agency, respectively) in personal work-identity formation.

Outcome	R^2^	Beta (β)	df	*F*	*t*	*p*
Coherence	.57		5, 761	198.41		<.001
		.20 Familiarity			7.90	.001
		-.01 Missing			- .50	.617
		.27 Bonding			7.90	<.001
		-.03 Proudness			-1.02	.308
		.51 Emotional Agency			15.33	<.001
Correspondence	.29		5, 761	62.91		<.001
		.07 Familiarity			2.10	.036
		.09 Missing			2.45	.014
		.17 Bonding			3.86	<.001
		-.13 Proudness			-3.41	<.001
		.41 Emotional Agency			9.78	<.001
Mental Time	.17		5, 761	31.29		<.001
		.04 Familiarity			1.19	.234
		.06 Missing			1.62	.106
		.09 Bonding			1.83	.068
		-.09 Proudness			- 2.28	.023
		.35 Emotional Agency			7.70	<.001
Reflection	.18		5, 761	33.98		<.001
		.08 Familiarity			2.26	.024
		.06 Missing			1.62	.106
		.14 Bonding			2.98	.003
		.01 Proudness			.24	.811
		.26 Emotional Agency			5.74	<.001
Cognitive Agency	.34		5, 761	79.54		<.001
		.05 Familiarity			1.64	.101
		.03 Missing			.99	.321
		.20 Bonding			4.79	<.001
		-.15 Proudness			-4.21	<.001
		.48 Emotional Agency			11.68	<.001

#### 4.1.2 Cognitive processes predicting emotional processes of collective work-identity

In line with Hypothesis 1b, the cognitive processes significantly predicted each of the three emotional processes in collective work-identity, with an explained variance of 33% for Proudness, 67% for Esteem and 29% for Affective Commitment. Assimilation showed the strongest positive association, while Incorporation and Identification showed mixed, although positive, results in associating with the three emotional processes of collective work-identity (see Table 5 for details).

**Table 5:** The cognitive processes (Incorporation, Identification and Assimilation) predicting the emotional processes (Proudness, Esteem and Affective Commitment, respectively) in collective work-identity formation.

Outcome	R^2^	Beta (β)	df	*F*	*t*	*p*
Proudness	.33		3,763	124.83		<.001
		.24 Incorporation			7.04	<.001
		.18 Identification			4.48	<.001
		.28 Assimilation			6.86	<.001
Esteem	.67		3,763	508.22		<.001
		.08 Incorporation			3.40	<.001
		.17 Identification			5.92	<.001
		.66 Assimilation			23.19	<.001
Affective Commitment	.29		3,763	102.71		<.001
		.21 Incorporation			5.97	<.001
		.10 Identification			2.49	.013
		.33 Assimilation			7.87	<.001

### 4.2 Person-oriented analysis

#### 4.2.1 Emotional profiles of personal work-identity

**Figure 3:**
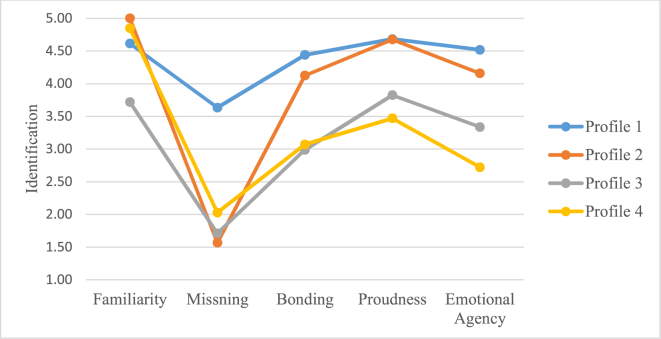
The four identity profiles along the emotional processes (Familiarity, Missing, Bonding, Proudness and Emotional Agency in mean values of identification) of the personal work-identity.

Given the four cell matrix (see Table 1) and the dendrogram, a four-cluster solution was obtained by a hierarchical cluster analysis showing four work-identity profiles based on the emotional processes (Familiarity, Missing, Bonding, Proudness and Emotional agency) of personal work-identity. The four work-identity profiles (depicted in Figure 3, see Table 6 for details) were as follows: Profile 1 consisted of individuals labeled as “Strong and Consistent Emotionals”; Profile 2 consisted of individuals labeled as “Strong but Moderate Inconsistent Emotionals; Profile 3 consisted of individuals labeled as “Moderate Strong and Moderate Consistent Emotionals”; Profile 4 consisted of individuals labeled as “Moderate Strong but Moderate Inconsistent Emotionals”.

**Table 6:** Descriptive statistics for each of the four profiles based on the emotional processes of personal work-identity; demographics [N, age, males, females, permanent employment (PE), mean part of full time employment (PFTE), years of employment (YE), non-public working sector (NP sector), public working sector (P sector)] and means (and SD) for each of the profiles along the emotional processes of personal work-identity (E-PWI).

Outcome	Profile 1	Profile 2	Profile 3	Profile 4
**Demographics**				
N (numbers and %)	218 (28%)	120 (16%)	246 (32%)	183 (24%)
Age (mean)	45.30	51.00	43.80	47.70
Males (numbers and %)	32 (15%)	33 (27%)	72 (29%)	51 (28%)
Females (numbers and %)	186 (85%)	87 (73%)	174 71%)	132 (72%)
PE (numbers and %)	216 (99%)	119 (99%)	221 (90%)	174 (95%)
PFTE (% of full-time employment)	96.33	95.93	93.60	94.05
YE (mean)	14.05	17.50	11.28	15.17
NP sector (numbers and %)	206 (94%)	112 (93%)	221 (90%)	170 (93%)
P sector (numbers and %)	12 (6%)	8 (7%)	25 (10%)	13 (7%)
**E-PWI processes**				
Familiarity	4.61 (0.49)	5.00 (0.00)	3.72 (0.61)	4.85 (0.36)
Missing	3.63 (0.73)	1.57 (0.56)	1.71 (0.75)	2.03 (1.00)
Bonding	4.44 (0.56)	4.13 (0.78)	2.99 (0.97)	3.07 (1.02)
Proudness	4.68 (0.49)	4.68 (0.47)	3.83 (0.85)	3.47 (1.10)
Emotional Agency	4.52 (0.55)	4.16 (0.67)	3.34 (1.00)	2.72 (0.89)

#### 4.2.2 Emotional profile effects on cognitive processes of personal work-identity

In line with Hypothesis 2a, the multivariate analyses showed an overall effect of work-identity profile (based on the emotional processes) on the cognitive processes of personal work-identity, Wilks’ Lambda (Λ) = .64, *F* (5, 759) = 24.29, *p*= <.001, *ηρ*^*2*^ *=* .14.

The univariate analysis showed effects of work-identity profile (based on the emotional processes) on each of the cognitive processes of personal work-identity with a *ηρ*^*2*^ of .33 for Coherence, .17 for Correspondence, .10 for Mental Time, .12 for Reflection and .17 for Cognitive Agency, see Table 7 for details. Post hoc comparisons showed that Profile 1 (“Strong and Consistent Emotionals”) and Profile 2 (“Strong but Moderate Inconsistent Emotionals”), respectively scored higher on each of the five cognitive processes compared to Profile 3 (“Moderate Strong and Moderate Consistent Emotionals”) and Profile 4 (“Moderate Strong but Moderate Inconsistent Emotionals”), respectively, see Tables 7 and 8 for details. There were no differences between Profiles 1 and 2, between Profiles 3 and 4 in any of the five cognitive processes of personal work-identity, except for Cognitive Agency, where profile 3 and 4 differed significantly, see Table 8 for details.

**Table 7:** Univariate analyses of the effects of work-identity profiles (based on the emotional processes) on the cognitive process (Coherence, Correspondence, Mental Time, Reflection and Cognitive Agency, respectively) of personal work-identity.

Outcome	Profile	Mean (SD)	*F*	df	*ηρ*^*2*^	*p*
Coherence			122.80	3, 763	.33	<.001

	1	4.32 (.82)				
	2	4.22 (.69)				
	3	3.03 (1.08				
	4	2.90 (1.00)				

Correspondence			53.54	3, 763	.17	<.001

	1	3.90 (1.02)				

	2	3.63 (1.08)				
	3	2.93 (1.05)				
	4	2.72 (1.16)				

Mental time			27.61	3, 763	.10	<.001

	1	4.14 (.98)				
	2	3.84 (1.12)				
	3	3.39 (1.10)				
	4	3.28 (1.17)				
Reflection			33.61	3, 763	.12	<.001

	1	4.33 (.80)				
	2	4.15 (.85)				
	3	3.64 (.94)				
	4	3.62 (.92)				
Cognitive Agency			53.22	3, 763	.17	<.001

	1	4.22 (.83)				
	2	4.09 (.85)				
	3	3.42 (1.03)				
	4	3.16 (1.11)

**Table 8:** Multiple comparisons between the four profiles of emotional personal work-identity for each of the cognitive processes of personal work-identity, using Bonferroni post-hoc comparisons.

Outcome	Profile (I)	Profile (J)	Mean difference (I-J)	*p*
Coherence	1	2	.10	1.000
		3	1.28	<.001
		4	1.41	<.001
	2	3	1.18	<.001
		4	1.32	<.001
	3	4	.13	.920
Correspondence	1	2	.28	.140
		3	.97	<.001
		4	.1.18	<.001
	2	3	.69	<.001
		4	.90	<.001
	3	4	.21	.280
Mental time	1	2	.30	.100
		3	.75	<.001
		4	.86	<.001
	2	3	.45	.001
		4	.56	<.001
	3	4	.11	1.000
Reflection	1	2	.18	.440
		3	.69	<.001
		4	.71	<.001
	2	3	.51	<.001
		4	.53	<.001
	3	4	.02	1.000
Cognitive Agency	1	2	.13	1.000
		3	.81	<.001
		4	1.06	<.001
	2	3	.67	<.001
		4	.93	<.001
	3	4	.25	.044

#### 4.2.3 Cognitive profiles of collective work-identity

**Figure 4:**
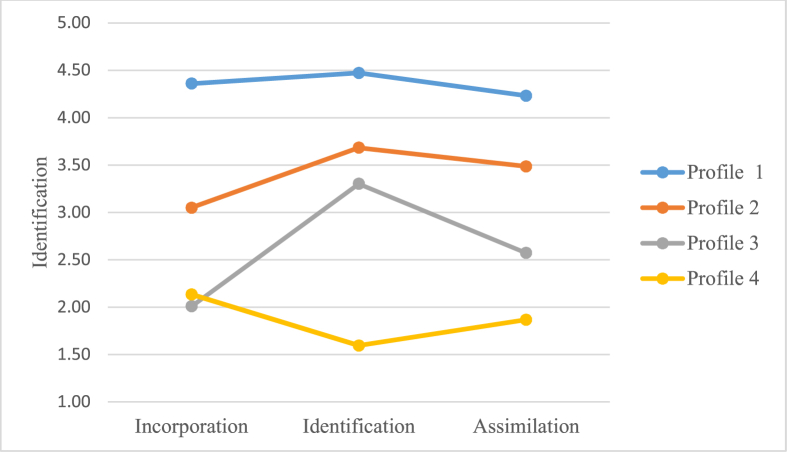
The four identity profiles along the cognitive processes (Incorporation, Identification and Assimilation in mean values of identification) of the collective work-identity.

Given the four cell matrix (see Table 1) and the dendrogram, a four-cluster solution was obtained by the hierarchical cluster analysis, showing four work-identity profiles based on the cognitive processes (Incorporation, Identification and Assimilation) of collective work-identity. The four work-identity profiles (depicted in Figure 4, see Table 9 for details) were as follows: Profile 1 consisted of individuals labeled as “Strong and Consistent Identifiers”; Profile 2 consisted of individuals labeled as “Moderate Strong and Consistent Identifiers”; Profile 3 consisted of individuals labeled as “Moderate Weak and Moderate Inconsistent Identifiers”; Profile 4 consisted of individuals labeled as “Weak but Consistent Identifiers”.

**Table 9:** Descriptive statistics for each of the four profiles based on the cognitive processes of collective work-identity; demographics [N, age, males, females, permanent employment (PE), mean part of full time employment (PFTE), years of employment (YE), non-public working sector (NP sector), public working sector (P sector)] and means (and SD) for each of the profiles along the cognitive processes of collective work-identity (C-CWI).

Outcome	Profile 1	Profile 2	Profile 3	Profile 4
**Demographics**				
N (numbers and %)	125 (16%)	284 (37%)	126 (16%)	232 (30%)
Age (mean)	45.81	45.86	46.38	46.97
Males (numbers and %)	19 (15%)	72 (25%)	33 (26%)	64 (28%)
Females (numbers and %)	106 (85%)	212 (75%)	93 (74%)	168 (72%)
PE (numbers and %)	121 (97%)	276 (97%)	120 (95%)	213 (92%)
PFTE (% of full-time employment)	97.00	95.50	92.00	94.50
YE (mean)	13.50	14.20	12.90	14.53
NP sector (numbers and %)	115 (92%)	263 (93%)	116 (92%)	215 (93%)
P sector (numbers and %)	10 (8%)	21 (7%)	10 (8%)	17 (7%)
**C-CWI processes**				
Incorporation	4.36 (0.48)	3.05 (0.71)	2.01 (0.68)	2.13 (0.94)
Identification	4.47 (0.55)	3.68 (0.84)	3.30 (0.65)	1.59 (0.55)
Assimilation	4.23 (0.58)	3.49 (0.79)	2.57 (0.84)	1.87 (0.74)

#### 4.2.4 Cognitive profile effects on emotional processes of collective work-identity

In line with Hypothesis 2b, the multivariate analysis showed an overall effect of the work-identity profiles (based on the cognitive processes) on the emotional processes of collective work-identity, Wilks’ Lambda (Λ) = .50, *F* (3, 761) = 67.05, *p*= <001, *ηρ*^*2*^ *=* .20.

The univariate analyses showed effects of the work-identity profiles (based on the cognitive processes) on each of the emotional processes of collective work-identity with a *ηρ*^*2*^ of .26 for Proudness, .47 for Esteem and .22 for Affective commitment, see Table 10 for details. Post-hoc comparisons showed that Profile 1 (“Strong and Consistent Identifiers”) scored highest, followed by Profile 2 (“Moderate Strong and Consistent Identifiers”), followed by Profile 3 (“Moderate Weak and Moderate Inconsistent Identifiers”), followed by Profile 4 (“Weak but Consistent Identifiers”) in each of the three emotional processes of collective work-identity, see Tables 10 and 11 for details.

**Table 10:** Univariate analyses of the effects of work-identity profiles (based on the cognitive processes) on the emotional process (Proudness, Esteem and Affective Commitment, respectively) of collective work-identity.

Outcome	Profile	Mean (SD)	*F*	df	*ηρ*^*2*^	*p*
Proudness			91.10	3, 763	.26	<.001

	1	3.25 (1.27)				
	2	2.59 (1.00)				
	3	1.92 (.90)				
	4	1.61 (.84)				
		Esteem	222.84	3, 763	.47	<.001

	1	3.94 (.82)				
	2	3.14 (.92)				
	3	2.40 (.82)				
	4	1.74 (.80)				
Affective commitment			70.65	3, 763	.22	<.001

	1	3.70 (1.20)				
	2	3.05 (1.10)				
	3	2.45 (1.20)				
	4	2.04 (1.11)

**Table 11:** Multiple comparisons between the four profiles of cognitive collective work-identity for each of the emotional processes of collective work-identity, using Bonferroni post-hoc comparisons.

Outcome	Profile (I)	Profile (J)	Mean difference (I-J)	*p*
Proudness	1	2	.66	<.001
		3	1.33	<.001
		4	1.64	<.001
	2	3	.67	<.001
		4	.98	<.001
	3	4	.31	.030
Esteem	1	2	.80	<.001
		3	1.54	<.001
		4	2.20	<.001
	2	3	.74	<.001
		4	1.40	<.001
	3	4	.66	<.001
Affective commitment	1	2	.65	<.001
		3	1.24	<.001
		4	1.65	<.001
	2	3	.59	<.001
		4	1.00	<.001
	3	4	.41	.008

## 5 Discussion

We investigated emotion and cognition involved in formation of personal and collective work-identity by variable- and person-oriented analyses, encompassing two theoretical perspectives of personal autobiographical memory and social identity. This is crucial given that personal work-identity formation relates to an autobiographical memory perspective (see Conway et al., 2004; Klein et al., 2004; Knez, 2014; 2016; 2017; Knez and Nordhall, 2017; Knez et al., 2017; Knez et al., 2019), whereas formation of collective work-identity emanates from a social identity view (see Ashforth and Mael, 1989; Hogg, 2012). We applied the two different types of analyses because: (1) a variable-oriented analysis treats processes as separated factors/entities investigating linear relationships between these factors, and (2) a person-oriented analysis implies a holistic perspective, treating individuals as different clusters with different profiles (i.e. combinations) of a set of factors e.g. processes. By combining a variable- and a person-oriented view of analysis it is possible to attain a broader perspective and a deeper understanding of, e.g. work-identity formation. This is important, because it yields information about the contribution of the different and separate psychological processes involved, as well as the contribution of individual combinations of such processes, in work-identity formation (see Bergman, 2000; Bergman et al., 2000; Magnusson, 2000 for this type of argument).

Previous theoretical and empirical accounts have indicated personal work-identity formation as the emotion component/processes, temporally preceding and affecting the cognitive component/processes (see Knez, 2014; 2016). Also, positive relationships between personal work-identity and outcomes of wellness and mental health have been accounted for by the emotion component (see Nordhall and Knez, 2018; Nordhall et al., 2018). In contrast to personal work-identity formation, the cognition component/processes temporally may precede and affect the emotion component/processes in collective work-identity formation (Mael and Ashforth, 1992; Van Knippenberg and Sleebos, 2006). Also, the cognition component have been shown to account for the positive relation between collective work-identity and outcomes of wellness and mental health (see Nordhall and Knez, 2018; Nordhall et al., 2018).

In line with our prediction H1a, and an autobiographical memory perspective (see Conway et al., 2004; Klein et al., 2004; Knez, 2016), the results show that, by a variable-oriented analysis, the emotional processes of familiarity, missing, bonding, proudness and emotional agency overall positively predicted the cognitive processes of coherence, correspondence, mental time, reflection and cognitive agency. More specifically, the results of H1a indicate that when, above all, the process of emotional agency (work as part of oneself) increases, the cognitive personal work-bonding also increases, primarily in terms of coherence (continuity in the self-work relation across time). However, the emotional processes of proudness (of ones work) negatively or not at all associated with the cognitive processes in personal work-related bonding. This indicates that proudness as a psychological process have a different role in personal work-identity formation in that stronger feelings of proudness of one's personal work implies weaker cognitive person-work bonding or even no changes at all in this type of psychological work-bonding. Also, the process of missing (of ones work) seems to be of less importance in that it overall was not related to the cognitive processes of personal work-identity. Additionally, using a person-oriented analysis, the results concerning H2a tentatively indicate effects of emotional profile on cognitive processes in personal work-identity formation. More specifically, the results concerning H2a show that those individuals that profile as, “strong and consistent emotionals” or “strong but moderate inconsistent emotionals” in their emotional personal work-bonding, also show stronger cognitive personal work-bonding than those individuals profiling as “moderate strong and moderate consistent emotionals” or “moderate strong but moderate inconsistent emotionals”.

In line with an autobiographical memory perspective (e.g., Knez, 2014; 2016), the personal work-identity formation, involving the individual self (Brewer and Gardner, 1996), implies a need to *distinguish* oneself from others by obtaining a personal self-definition and interpretation in a working context in order to maintain the story and memories of the personal self (see Knez 2016; Nordhall et al., 2018). The present results concerning H1a indicate that such a need and work-related self-definition to a larger extent involve the emotional process of emotional agency (work as part of oneself) and the cognitive process of coherence (continuity in the self-work relation across time). In accordance with the results of H2a, such self-definition may vary between individuals due to their emotional profile in the personal work-identity. This, primarily in terms of strength (and less in terms of consistency) in the emotional personal work-bonding (see Canrinus et al., 2011; Endedijk et al., 2017; Groth et al., 2017; Pillen et al., 2013).

Regarding emotion and cognition in collective work-identity formation, in line with our prediction H1b and a social identity perspective (see Ashforth and Mael, 1989; Hogg, 2012), the results show that, by a variable-oriented analysis, the cognitive processes of incorporation, identification and assimilation positively predicted the emotional processes of proudness, esteem and affective commitment. More specifically, these results show that when, above all, the cognitive process of assimilation (of organizational successes) increases, the emotional collective work-bonding, primarily in terms of esteem (of organizational belonging) also increases. Additionally, using a person-oriented analysis, the results for H2b tentatively indicate effects of cognitive profile on emotional processes in collective work-identity formation. More specifically, the results for H2b show that those individuals profiling as “strong and consistent identifiers” in the cognitive collective work bonding, also show the strongest emotional collective work bonding. Individuals that profile as “weak but consistent identifiers” show the weakest emotional collective work bonding.

In line with a social identity (e.g. Ashforth and Mael, 1989) and self-categorization (e.g., Turner et al., 1987) perspective, collective work-identity formation implies a need of *belonging* to a certain group involving the definition of a social/collective self (Brewer and Gardner, 1996). Such a collective self-definition and categorization implies a depersonalization of the individual self and the knowledge of being part of a group/organization (Tajfel and Turner, 1979; 1986; see Hogg, 2012 for a review) as well as the collective self, the collective story and its memories (Knez, 2016). The results of H1b indicate that such a need and work-related self-definition to a larger extent involve cognitive process of assimilation (of organizational successes) and the emotional process of esteem (of organizational belonging). Also, and in accordance with the results of H2b, such collective self-definition may vary between individuals due to their cognitive profile in the collective work-identity. This, mostly in terms of strength (and less in terms of consistency) in the cognitive collective work-bonding (see Canrinus et al., 2011; Endedijk et al., 2017; Groth et al., 2017; Pillen et al., 2013).

Insights into emotional and cognitive processes and profiles involved in work-identity formation may be of some value also in the context of organizational change, which involves role identity transitions that may imply changing or differentiation in work roles (see Ashforth, 2001; Oreg et al., 2013; Stets and Burke, 2000). Organizational change has been shown to challenge, and be challenged, by a strong work-identity and it may be difficult to force a change in work-identity. Reorganization and transitions of work roles during organizational changes may imply a psychological break up of prevailing work identifications as well as reformation of new work-identities, entailing the individual employee experiencing a loss of control, thereby increasing his/her stress-reactions (Drzensky and Van Dick, 2013). In view of this, emotional and cognitive work-identity processes and profiles may have implications for how the individual employee is affected by the organizational change and for his/her adaption of the aftermaths. Also, insights into, and reformations of emotion and cognition in work-identification may enhance the fit between the individual employee (work-identity profile) and occupational work during and after an organizational change. This may enhance organizational effectiveness as well as employees’ well-being and mental health (for similar views and practices, see Pellegrini et al., 2018; Zappal et al., 2019).

Finally, some limitations of the present study should be mentioned. First, the results are based on cross-sectional data, and by that lacking random assignment. Thus, it is not possible to draw definite conclusions about causation/development over time. However, we have reported data on the *momentary set up* of work-identity in subjects with a mean employment time of 14 years. This means that the work-identity formation was investigated relatively the period of employment, proposing that the longer employment time the more the *momentary set up* of work-identity will correspond to the work-identity formation measured across time. Accordingly, our data suggest how work-identity formation may develop across the employment. Also, the present results are in line with previous theoretical and empirical accounts suggesting causal relationships along a temporal dimension (see e.g. Knez et al. 2018a for this type of argument).

Second, the response rate of 26% may be regarded as low, potentially reducing the opportunities for more general conclusions. However, nearly 800 participants, teachers representative of the Swedish teacher population, participated in the present study, which is satisfying for the statistics used (Tabachnick and Fidell, 2012). In addition, our hypotheses were based on general theoretical accounts and previous empirical findings, and the results obtained showed to be congruent with these standpoints. Third, we did not include any educational and/or school-related factors in our analyses because the aim was to investigate general relationships between the phenomena involved, exemplified by a sample of teachers.

## 6 Conclusions

Overall and concerning the *momentary set up* of work-identity formation, our results suggest: (1) For personal work-identity formation; emotion positively predicts cognition. (2) For collective work-identity formation; cognition positively predicts emotion. (3) These associations may vary between individuals due to their profile (i.e. combination) along the emotional personal- and cognitive collective work-bonding.

By this, emotion and cognition seem to play different roles in personal- and collective work-identity formation and thus the phenomenon of people-work bonding is a complex vocational construct. In order to grasp it, future research might involve both emotional- and cognitive processes of work-identification within personal and social theory perspectives as well as analyses at different levels (person- and variable oriented).

The practical implications of the results obtained are: knowledge of emotional and cognitive processes and profiles in work-identity formation might be useful for organizations in their general work on human resource policies and during reorganizations. This in order to attain a more optimal fit between the occupational work and the individual employee which may enhance the organizational effectiveness as well as employees’ well-being and mental health.

## Declarations

### Author contribution statement

Ola Nordhall: Conceived and designed the experiments; Performed the experiments; Analyzed and interpreted the data; Contributed reagents, materials, analysis tools or data; Wrote the paper.

Igor Knez, Johan Willander: Conceived and designed the experiments; Analyzed and interpreted the data; Wrote the paper.

### Funding statement

This research did not receive any specific grant from funding agencies in the public, commercial, or not-for-profit sectors.

### Data availability statement

Data will be made available on request.

### Declaration of interests statement

The authors declare no conflict of interest.

### Additional information

Supplementary content related to this article has been published online at Data In Brief.
